# Near-Road Traffic-Related Air Pollution: Resuspended PM_2.5_ from Highways and Arterials

**DOI:** 10.3390/ijerph17082851

**Published:** 2020-04-21

**Authors:** Mohammad Hashem Askariyeh, Madhusudhan Venugopal, Haneen Khreis, Andrew Birt, Josias Zietsman

**Affiliations:** 1 Zachry Department of Civil and Environmental Engineering, College Station, TX 77843-3127, USA; 2 Environment and Air Quality Division, Texas A&M Transportation Institute, College Station, TX 77843-3135, USA; m-venugopal@tti.tamu.edu (M.V.); a-birt@tti.tamu.edu (A.B.); 3 Center for Advancing Research in Transportation Emissions, Energy, and Health (CARTEEH), Texas A&M Transportation Institute, College Station, TX 77843-3135, USA; h-khreis@tti.tamu.edu (H.K.); zietsman@tamu.edu (J.Z.)

**Keywords:** resuspended dust, road dust, PM_2.5_, near-road, traffic, air pollution, MOtor Vehicle Emission Simulator (MOVES), American Meteorological Society/Environmental Protection Agency Regulatory Model (AERMOD)

## Abstract

Recent studies suggest that the transportation sector is a major contributor to fine particulate matter (PM_2.5_) in urban areas. A growing body of literature indicates PM_2.5_ exposure can lead to adverse health effects, and that PM_2.5_ concentrations are often elevated close to roadways. The transportation sector produces PM_2.5_ emissions from combustion, brake wear, tire wear, and resuspended dust. Traffic-related resuspended dust is particulate matter, previously deposited on the surface of roadways that becomes resuspended into the air by the movement of traffic. The objective of this study was to use regulatory guidelines to model the contribution of resuspended dust to near-road traffic-related PM_2.5_ concentrations. The U.S. Environmental Protection Agency (EPA) guidelines for quantitative hotspot analysis were used to predict traffic-related PM_2.5_ concentrations for a small network in Dallas, Texas. Results show that the inclusion of resuspended dust in the emission and dispersion modeling chain increases prediction of near-road PM_2.5_ concentrations by up to 74%. The results also suggest elevated PM_2.5_ concentrations near arterial roads. Our results are discussed in the context of human exposure to traffic-related air pollution.

## 1. Introduction

In recent years, there has been a focus on the adverse health effects of near-road long- and short-term exposure to traffic-related air pollutants [1,2,3,4,5,6]. Fine particulate matter (PM_2.5_) is a U.S. Environmental Protection Agency (EPA) regulated criteria air pollutant [7]. PM_2.5_ is emitted from different emission sources including the transportation sector, and studies have demonstrated elevated PM_2.5_ concentrations in near-road environments [8,9,10,11,12]. A growing body of literature shows associations between higher exposure to PM_2.5_, due to proximity of residential areas to major roadways and adverse health effects [13,14,15,16]. Approximately 11%–19% of the U.S. population live within a few hundred meters of major roads [15,17,18], which can affect more than 40 million people exposed to high levels of PM_2.5_ in the U.S. The global population exposed to elevated levels of PM_2.5_ is far greater. Hence, monitoring and modeling transportation-related PM_2.5_ emissions and their dispersion are important for understanding human exposure and health risks. For example, emission and dispersion modeling of PM_2.5_ is a requirement of regulatory quantitative analyses for federally supported new transportation projects in nonattainment and maintenance areas [19]. The PM_2.5_ emissions from the transportation sector result from tailpipe exhaust, brake wear, tire wear, and resuspended dust and are explained in the U.S. EPA-developed MOtor Vehicle Emission Simulator (MOVES) guidance and transportation conformity guidance for PM_2.5_ quantitative hotspot analysis [19,20]. The objective of this study was to use these regulatory guidelines to quantify the contribution of resuspended dust compared to other traffic-related PM_2.5_ concentrations from arterials and highways.

The tailpipe exhaust component of PM_2.5_ emissions has decreased considerably as different exhaust emission control measures have been deployed [21]. However, current non-exhaust emissions from road vehicles are unabated, making the contribution of resuspended road dust to traffic-related particulate matters even more significant [22,23]. An intensive mass and chemical measurement included study showed that the PM_2.5_ emission rate from resuspended dust is significant and can exceed the tailpipe contribution in Reno, Nevada [24]. Kundu et al. compared the composition of PM_2.5_ in rural and urban areas and concluded that unpaved roads can contribute to a significantly higher level of PM_2.5_ at five sampling sites in Iowa [25]. Amato et al. performed an extensive field measurement study and showed that a poor state of pavement can double the road dust loading [22]. While these studies showed the importance of including resuspended PM_2.5_ in air pollution studies, other studies did not conclude that resuspended dust is a significant source of PM_2.5_; rather, on-road emission sources were more significant [26].

In emission and dispersion modeling for regulatory purposes, a procedure including specific guidelines to estimate PM_2.5_ emissions from transportation and perform dispersion modeling should be followed [19]. In this procedure, the PM_2.5_ emissions from tailpipe exhaust, brake wear, tire wear, and resuspended dust emissions should also be modeled. The EPA’s MOVES2014a on-road model and current AP-42 paved road resuspended dust model are recommended to estimate PM_2.5_ emission rates (PM_2.5_ mass per time) as per the hotspot guidance [19,27,28]. While tailpipe exhaust (running, idling, and start), brake, and tire wear are estimated using EPA’s MOVES modeling tool [20], resuspended dust calculations utilize AP-42 factors which have gone through limited updates. MOVES is an emission model that uses a fine-scale modal-based approach to generate emission and energy consumption factors at different temporal geographical scales (national, county, and project) [20].

The emission factors obtained from the MOVES can be used with transportation activity to estimate total emissions from all roadway links. For project level emissions assessment, MOVES requires inputs from two broad categories: (a) site-specific traffic information, including traffic volumes, fleet composition, and vehicle activity at the roadway link level, and (b) local-specific inputs, including regional-level vehicle age distribution, meteorological variables, fuel characteristics, and parameters related to the inspection/maintenance (I/M) program. The modeled emission rates can then be used for dispersion modeling to consider the effect of meteorological variables and predict near-road PM_2.5_ concentrations. Air pollutant dispersion for regulatory purposes needs to be modeled using the American Meteorological Society/Environmental Protection Agency Regulatory Model (AERMOD) [19,29]. AERMOD is a Gaussian steady-state dispersion model that predicts the concentration of air pollutants emitted from characterized emission sources.

Many different studies conducted around the world have shown the effect of resuspended road dust on traffic-related PM_2.5_ emissions. However, the effect of using a network with and without road-dust on the dispersion models’ predictions of near-road PM_2.5_ concentrations is a less investigated area. In addition, the sensitivity of regulatory quantitative analysis to resuspended PM_2.5_ on highways and arterials has not been investigated. Previous studies have shown a nonlinear relationship between emission rates and near-road air pollutants concentrations over different time periods due to the effect of meteorological variables on dispersion mechanisms [30,31,32]. For instance, a constant emission rate yields different concentrations under different meteorological conditions over time [8]. However, the effects of a certain change in PM_2.5_ emissions due to the inclusion of resuspended road dust on near-road PM_2.5_ concentrations over different time periods have not been evaluated.

The objective of this study was to quantify the contribution of highways and arterials resuspended dust to the traffic-related PM_2.5_ emissions and near-road concentrations, following the U.S. EPA regulatory guidelines. As such, the increase in PM_2.5_ emission rates due to the inclusion of resuspended road dust in Fort Worth, Texas was investigated, following transportation conformity guidance for PM_2.5_ quantitative hotspot analysis. Additionally, the dispersion modeling was performed using a 2016 dataset of monitored meteorological variables to evaluate the sensitivity of predicted traffic-related PM_2.5_ concentrations in a near-road environment at different seasonal day time periods. This study quantifies the sensitivity of dispersion modeling to a significant increase in PM_2.5_ emission rates in a network due to the inclusion of resuspended PM_2.5_.

## 2. Materials and Methods

### 2.1. Theoretical Premise

The road dust PM_2.5_ emission rate is a function of vehicle weight, road type (paved vs. unpaved), and meteorological variables (precipitation) which will be multiplied by traffic volume for resuspended dust emissions estimation. The PM_2.5_ emissions from tailpipe exhaust, brake wear and tire wear (exhaust and other) are functions of traffic speed, road type, fleet characteristics and mix, fuel quality, and meteorological variables in the respective county which will be multiplied by traffic volume for vehicular (exhaust, brake, and tire wear) emissions estimation [19]. Adding the resuspended PM_2.5_ emissions to the vehicular (exhaust, brake, and tire wear) PM_2.5_ emissions will be influenced by meteorological variables such as temperature, wind speed, and wind direction as a function of time when dispersion mechanisms occur [19,29]. In addition, adding the resuspended PM_2.5_ emission rate to various segments of a network with associated PM_2.5_ emission rates (due to vehicular sources with different characteristics and speeds) cannot be interpreted as a linear increase of the total PM_2.5_ emission rate in a network. Hence, it can be concluded that there is a nonlinear relationship between traffic-related emission rates and near-road traffic-related air pollutant concentrations over different time periods [12]. Changes in emission rates will not necessarily result in the same changes in concentrations. In this study, the traffic-related PM_2.5_ emissions (mass per time per area of roadway) and near-road traffic-related concentrations (mass per volume), were estimated for different time periods in 2016 for each of the four seasons, for two types of roadways (highways and arterials). Finally, the emission rates and predicted near-road concentrations were averaged over daily time periods for each season to compare the effects including or not including resuspended PM_2.5_ emissions. Through this procedure, the increment of near-road traffic-related PM_2.5_ concentrations including resuspended PM_2.5_ in traffic-related emissions was investigated.

### 2.2. Area of Study

**Figure 1:**
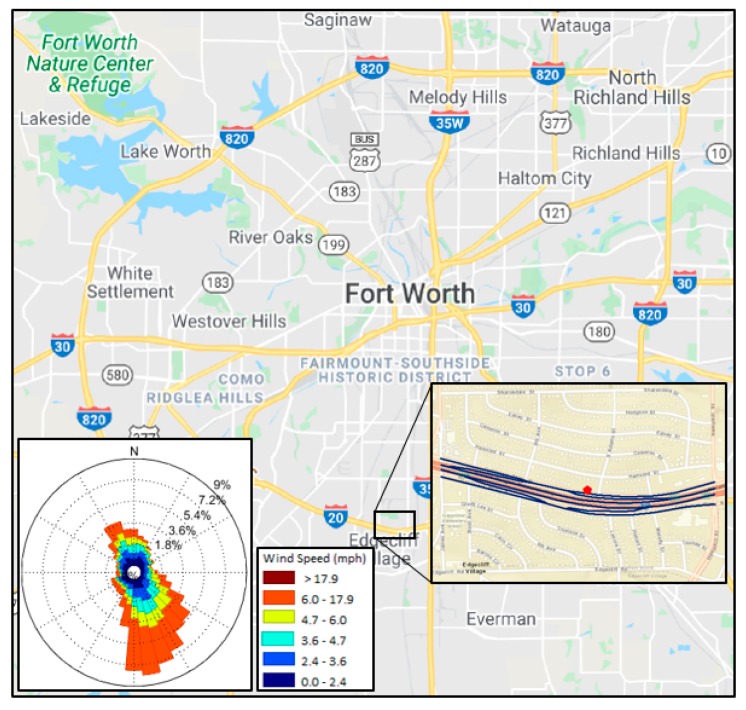
Study area (I-20: Ronald Reagan Memorial Highway shown by navy lines), near-road environment (shown by red mark) and corresponding wind rose based on monitored values in Fort Worth, Texas.

In response to recent EPA requirements for near-road air pollution monitoring, the Texas Commission on Environmental Quality (TCEQ) has determined six locations near major highways to monitor air quality using the federal reference method (FRM) in Texas [33]. One of these is in Fort Worth, Texas, and this location was selected for emission, meteorological, and dispersion modeling in this study [34]. The near-road continuous air monitoring station (CAMS) 1053 is located 15 m away from the edge of I-20 in Tarrant County (EPA Site Number: 484391053, 1198 California North, TX 76115, USA), as shown in Figure 1. The highway and arterial segments within a 600 m radius (*8*) of this near-road point (Figure 1) were considered for dispersion modeling. The hourly wind speed, wind direction, and temperature monitored at this point (CAMS 1053) were used as the onsite meteorological data for data processing in the meteorological modeling.

The Dallas-Fort Worth (DFW) regional travel demand model (TDM) results were obtained from the North Central Texas Council of Governments and post-processed to estimate hourly traffic activity on each link for the target area. The modeled hourly traffic volume and speed were mapped into different daily time periods such as morning peak (6:00–9:00 a.m.), midday (9:00 a.m.–4:00 p.m.), evening peak (4:00–7:00 p.m.), and overnight (8:00 p.m.–6:00 a.m.) periods. The hour that corresponds to the maximum volume in each period was selected for the analysis. Traffic volume and speed were not adjusted for different seasons.

### 2.3. Emission Modeling Using MOVES

The PM_2.5_ emission factors due to all traffic-related sources other than resuspended dust (exhaust, brake, and tire wear) were modeled using MOVES for Tarrant County in 2016. MOVES requires information for vehicle types, ages, fuel types, and the emission parameters to estimate emission factors. To estimate composite emission factors for each link in the target network, the vehicle miles traveled (VMT) mix was obtained for two road types: highways and arterials.

The VMT mix indicates the contribution of each vehicle type to the total VMT. The VMT mixes were estimated using a previously developed method and expanded to produce the four daily time period estimates for four months [35,36]. The four daily time periods included morning peak, midday, evening peak, and overnight. The four months were January, April, July, and October and represent emissions in winter, spring, summer, and fall, respectively. Composite emission factors were estimated using MOVES emission factors for different vehicle types and VMT mix for two road types (arterials and highways) based on Equation (1) [35], in which *i* represents vehicle types.

(1) $$Composite\;EF=\underset{i}{\sum }Emission\;Factors\times VMT\;mix\;$$

### 2.4. Resuspended Dust Emission Estimation

No unpaved road emissions factor analyses were performed because there were no unpaved roads in the target network. Resuspended dust emission factors from paved roads (i.e., TDM and intra-zonal links) were developed according to Equation (2) from the AP-42 section 13.2.1 [28].

(2) $$E=k\;\left(sL^{0.91}\right)\left(W^{1.02}\right)\left(1-\frac{P}{4N}\right)$$

where *k* is the particulate size multiplier (g/VMT); *sL* is the road surface silt loading (g/m^2^); *W* is the average vehicle weight (tons); *P* is the number of wet days (≥0.01 inches of rain) (days); *N* is the number of days in the period (days).

The input parameters to estimate resuspended PM_2.5_ emissions are the PM_2.5_ particle size multiplier, a factor indicating road surface silt loading, the average weight of fleet, days with 0.01 inches or more precipitation (wet days) for the seasonal period, and number of days in the seasonal averaging period. The number of wet days for the seasonal periods of Tarrant County (37, 28, 29, and 13 days for spring, summer, fall, and winter, respectively) was obtained from the Community Collaborative Rain, Hail and Snow Network database [37]. The PM_2.5_ particle size multiplier (k = 0.25 g/VMT) and road surface silt loading (sL) for arterials (0.062 g/m^2^) and highways (0.003 g/m^2^) from the referenced EPA AP-42 guidance were used [28]. The average vehicle weight values were estimated using the current Tarrant County VMT mix and respective MOVES vehicle types weights. Since control programs (i.e., street sweeping) affect the road surface silt loading and controlled silt loading values are not available, no control programs were included in the development of the resuspended PM_2.5_ emissions factors for this analysis.

### 2.5. Dispersion Modeling Using AERMOD

Dispersion modeling requires an input set including meteorological variables and emission source characteristics. The meteorological inputs were obtained from running a meteorological preprocessor developed by the EPA for regulatory dispersion modeling, AERMET [38]. The onsite data including wind speed, wind direction, and temperature obtained from hourly near-road monitoring (CAMS 1053) were incorporated with upper air data and surface air data for 2016. Surface characteristics including albedo, Bowen ratio, and also surface air and upper air representative station name for Tarrant County were obtained from TCEQ meteorological database for air dispersion modeling [39]. The surface air data of Dallas Fort Worth Airport (Station ID: 3927) was obtained from the National Oceanic Atmospheric Administration (NOAA) surface air database [40] and upper air data of Fort Worth (Station ID: 3990) was obtained from NOAA Radiosonde Database [41]. AERMET was run including these raw input sets to model meteorological variables in hourly time resolution for target near-road environments in 2016.

To model the target network as the emission source in AERMOD, the network highways and arterials were split into smaller segments (to represent the curvature of the roads) and were defined as the area sources of PM_2.5_ emissions. The PM_2.5_ quantitative hotspot analyses were used to define the details of area sources of emissions [19]. The release height and initial vertical dispersion coefficient were estimated based on the EPA’s guidance for each of the four daily time periods for arterial and highway segments (approximately 1.4 m and 1.3 m, respectively). The PM_2.5_ concentrations were modeled for one receptor located at 15 m from the edge of the highway (32.66° N, –97.34° W, elevation: 214.9 m) and 4 m from ground-level representing the near-road environment.

## 3. Results and Discussion

### 3.1. Traffic-Related PM_2.5_ Emission Rates on Highways and Arterials

**Figure 2:**
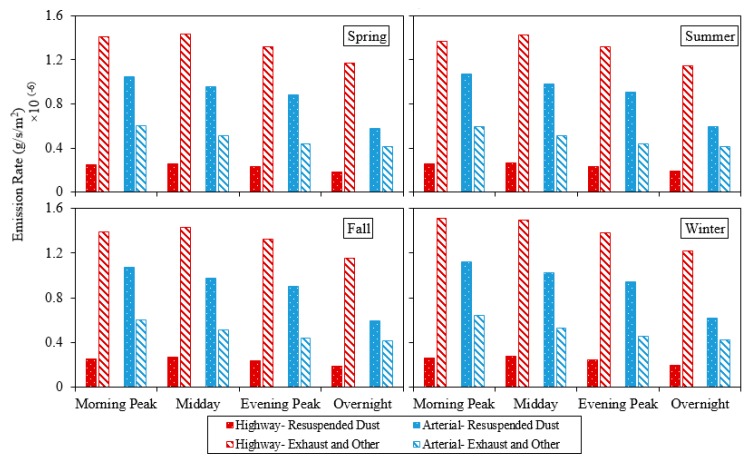
Predicted fine particulate matter (PM_2.5_) emission rates.

The PM_2.5_ emission rates due to resuspended dust and also exhaust emissions as averaged over four time periods of the day are shown for highways and arterials for four seasons in Figure 2. The predicted PM_2.5_ resuspended emissions are greater than exhaust, brake, and tire wear combined at arterials emphasizing the need to include resuspended dust in emission modeling when in close proximity to arterials. However, the resuspended PM_2.5_ emissions are significantly lower than exhaust, brake, and tire wear combined at highways which can be explained by the higher quality of highway pavement leading to smaller factors used in highway resuspended PM_2.5_ emission estimation.

The road surface silt loading (sL) plays a determinant role in the prediction of resuspended PM_2.5_ emissions from highways and arterials (0.003 and 0.062 g/m^2^, respectively, according to AP-42). Results do not show significant changes in emission rates between morning peak, midday, and evening peak, but a considerable decrease during nighttime due to the lower predicted traffic activity.

To investigate the PM_2.5_ emission rate increments due to inclusion of resuspended dust, the ratio of resuspended PM_2.5_ to the exhaust, brake, and tire wear emissions was calculated for highways and arterials in different seasonal and daily time periods (Table 1). The increments are consistently higher than 100% for arterials. For arterials, results also show that the increment is highest during evening peak followed by midday, morning peak, and overnight, respectively, which shows the importance of considering resuspended dust in PM_2.5_ emission estimation in the same order. Among different daily time periods for highway emissions, the increment is highest for midday, followed by morning peak, evening peak, and overnight, respectively. As far as seasonal variation, the increment is highest for summer, followed by fall, winter, and spring, respectively. The overall evaluation of modeled emission rates shows that the increase in average PM_2.5_, due to the inclusion of resuspended dust emissions, will vary from 15.7% to 18.7%, and 138.9% to 207.6% for highways and arterials in Fort Worth, Texas, respectively.

### 3.2. Traffic-Related PM_2.5_ Concentrations from Highways and Arterials

**Figure 3:**
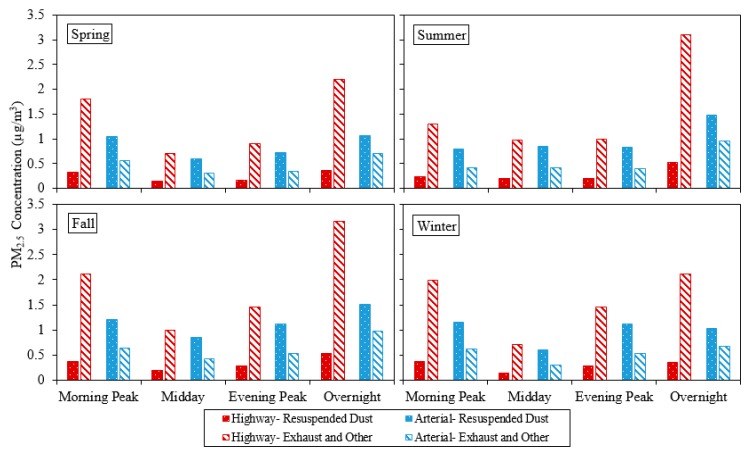
Predicted PM_2.5_ concentrations.

The PM_2.5_ emission rates were applied to the study network with a focus on highways and arterials to predict the average PM_2.5_ concentrations in four daily time periods of each season during 2016, as shown in Figure 3. In line with the emission results discussed above, the comparison of modeled concentrations shows a lower contribution of resuspended dust from highways and higher contribution from arterials when compared with exhaust, brake, and tire wear emissions. However, modeled PM_2.5_ concentrations resulting from traffic activity in highways and arterials show significant variation across the different daily time periods and the four seasons. This variation in PM_2.5_ concentrations is a result of various meteorological variables in different time periods caused by nonlinearity between traffic-related emissions and near-road concentrations over time, which cannot be detected by investigating daily and seasonal emission rates (Figure 2). The overnight traffic-related PM_2.5_ concentrations are typically highest, followed by those of morning peak, evening peak, and midday, respectively (Figure 3). The higher overnight near-road traffic-related PM_2.5_ is consistent with previous literature based on field [30] and modeling studies [8]. Previous research suggested that this trend may be due to the more stable/restrictive atmospheric boundary layer conditions during the night [42].

### 3.3. Overall Traffic-Related PM_2.5_ Concentrations

The near-road environment is located at different distances from various segments (highway and arterial with corresponding traffic count and speed), which comprise the whole target network and influence associated near-road traffic-related air pollution. The influence of different segments of the network on the target near-road environment depends on the geometry of the network and near-road environment, which will be combined with meteorological variables’ effect on emissions. This effect is the other source of nonlinearity between traffic-related emissions and near-road concentrations in dispersion modeling. Table 2 shows the increase in average near-road PM_2.5_ concentrations due to inclusion of resuspended dust emissions and dispersion modeling in different time periods and seasons.

The results show that adding resuspended PM_2.5_ emissions to the whole network (highways and arterials) yields significant increases (between 49% and 74.3%) in near-road traffic-related PM_2.5_ concentrations. The variation between different seasons is minimal. However, the increases are highest for midday and evening peak, followed by morning peak and overnight periods, respectively. The increments shown in Table 2 are different to those presented in Table 1 due to the nonlinearity of near-road traffic-related emission and concentration relationship due to the effect of meteorological variables and network geometry.

## 4. Conclusions and Recommendations

Resuspended dust is underinvestigated in the literature and is the component of traffic-related emissions which cannot be controlled by new technologies or new vehicle emission standards. Further, with the expected widespread introduction of electric vehicles (especially in cities), the relative importance of non-regulated, non-tailpipe emissions is becoming large. In the present study, the PM_2.5_ emission rates due to resuspended dust and exhaust, brake, and tire wear were modeled for two road types (highway and arterial), four time periods of the day, and four seasons in 2016, using EPA regulatory guidelines and tools. The increase in traffic-related PM_2.5_ emission and near-road concentrations due to inclusion of resuspended dust in estimations was evaluated and compared in different daily and seasonal time periods for a near-road environment in Tarrant County, Fort Worth, Texas. The estimated increase in traffic-related PM_2.5_ emissions was not proportional to the estimated near-road traffic-related PM_2.5_ concentrations at the different time periods. The nonlinearity between emission rates and concentrations due to the effect of meteorological variables and geometry of the network with unevenly scattered traffic-related emission rates (due to different link traffic speeds) was evident.

Increases in PM_2.5_ emission rates due to resuspended dust inclusion were considerably higher than the sum of tailpipe exhaust, brake wear, and tire wear emissions on arterials. The PM_2.5_ emission rate increments on arterials ranged between 139% and 208%, while they were lower on highways and ranged between 16% and 19%. The comparison of emission rates showed the importance of the inclusion of resuspended PM_2.5_, particularly when dealing with traffic-related PM_2.5_ in a near-road environment surrounded by arterials. These are areas where human exposure can be more important than areas near highways, as people tend to live, work, and congregate near many arterials. All PM_2.5_ emission rates overnight were lower than those modeled for three other daily periods during the year (which is expected due to the lower traffic counts), while modeled PM_2.5_ concentrations were highest overnight. The overall increase in near-road traffic-related PM_2.5_ concentrations for the whole network varied between 49% and 74%, an important percentage from an exposure and health point of view. A similar study using monitored hourly vehicle classification, traffic counts and speeds, and also near-road speciation data would be more reliable and useful for evaluation of regulatory guidelines in resuspended dust emission estimation and the exposure and health effect scenarios. In addition, the explained nonlinearity can be quantified using a monitored dataset and would be helpful to have a better understanding of influential variables and parameters in dispersion modeling. The study utilized AP-42 resuspended dust PM_2.5_ factors which has a rating of D [28] for application, this shows further studies are required to corroborate or update the existing AP-42 resuspended dust PM_2.5_ factors.

## Figures and Tables

**Table 1:** Predicted PM_2.5_ emission increment due to inclusion of resuspended dust.

Time Period	Highway				Arterial			
	Spring	Summer	Fall	Winter	Spring	Summer	Fall	Winter
Morning Peak	17.4%	18.4%	18.1%	17.5%	172.7%	181.3%	178.4%	175.6%
Midday	18.1%	18.7%	18.6%	18.6%	187.4%	193.0%	192.2%	193.1%
Evening Peak	17.3%	17.8%	17.8%	17.8%	202.1%	207.6%	206.8%	207.3%
Overnight	15.7%	16.4%	16.2%	16.2%	138.9%	144.1%	142.9%	144.8%

**Table 2:** Overall hourly average PM_2.5_ concentrations increment due to considering resuspended dust compared with those of a network without resuspended dust.

Time Period	Spring	Summer	Fall	Winter
Morning Peak	58.5%	60.2%	57.8%	58.3%
Midday	73.1%	73.8%	74.3%	73.3%
Evening Peak	72.2%	74.4%	71.1%	70.2%
Overnight	49.6%	49.4%	49.5%	49.8%
